# Radial Basis Functions Intended to Determine the Upper Bound of Absolute Dynamic Error at the Output of Voltage-Mode Accelerometers

**DOI:** 10.3390/s19194154

**Published:** 2019-09-25

**Authors:** Krzysztof Tomczyk, Marcin Piekarczyk, Grzegorz Sokal

**Affiliations:** 1 Faculty of Electrical and Computer Engineering, Cracow University of Technology, Warszawska 24, 31-155 Krakow, Poland; 2 Faculty of Mathematics, Physics and Technical Science, Pedagogical University of Cracow, 2 Podchorazych Ave, 30-084 Krakow, Poland; marcin.piekarczyk@up.krakow.pl (M.P.); grzegorz.sokal@up.krakow.pl (G.S.)

**Keywords:** radial basis function, upper bound of dynamic error, voltage-mode accelerometer

## Abstract

In this paper, we propose using the radial basis functions (*RBF*) to determine the upper bound of absolute dynamic error (*UAE*) at the output of a voltage-mode accelerometer. Such functions can be obtained as a result of approximating the error values determined for the assumed-in-advance parameter variability associated with the mathematical model of an accelerometer. This approximation was carried out using the radial basis function neural network (*RBF-NN*) procedure for a given number of the radial neurons. The Monte Carlo (MC) method was also applied to determine the related error when considering the uncertainties associated with the parameters of an accelerometer mathematical model. The upper bound of absolute dynamic error can be a quality ratio for comparing the errors produced by different types of voltage-mode accelerometers that have the same operational frequency bandwidth. Determination of the *RBFs* was performed by applying the Python-related scientific packages, while the calculations related both to the *UAE* and the MC method were carried out using the MathCad program. Application of the *RBFs* represent a new approach for determining the *UAE*. These functions allow for the easy and quick determination of the value of such errors.

## 1. Introduction

Acceleration, which is measured by accelerometers [1,2,3], is a feature of which instantaneous values are constantly changing [4]. A similar situation occurs when measuring other dynamic quantities (e.g., pressure, temperature, etc.) [5,6,7,8]. For those quantities, no explicit comparative criterion [9] has been developed so far, as is the case with the accuracy class of the instruments intended for static measurements [10,11]. When measuring acceleration, as is the case with other dynamic quantities [12,13,14,15], it is convenient to use the two best-known quality factors: The integral-square error [9,16,17] and the absolute error [12]. The first factor, for the assumed time of testing, allows one to determine the value of signal energy at the output of the sensor, while the second factor measures the maximum value of magnitude of this signal. However, both of these factors allow the determination of the numerical values of dynamic error for any measurement signal [9,12,13,14,15,17,18,19]. After the time corresponding to the time of the steady state of the sensor impulse response, the time characteristic of the integral-square error is linear [16,19], while the absolute error takes a constant value in time [12,18]. The time that it takes for the steady state of impulse response can be considered to be transient due to its very short duration.

Since the set of dynamic signals at the sensor input is infinite [9], in order to determine the upper bound of dynamic error [9,12,13,14] for the considered quality factor, the critical case of the input signal with limitations [20] should be determined. It can be carried out using the dedicated calculation algorithm intended for this purpose [12,13,14,15,18,19]. For the needs of such an algorithm, it is necessary to know the parameters of the mathematical model [2,3,4] of the considered sensor. Taking into account that from a practical point of view, only the error for the sensor’s operating band is of interest for the purposes of implementing such an algorithm, it is necessary to use the special standard as a reference to determine the error [9,12,21].

The main subject of this paper is the absolute dynamic error (*UAE*) for the absolute error criterion [12]. It is produced by the signal with limitations on both the magnitude and its duration [16,20]. An example of testing a voltage-mode accelerometer was considered in this work. It was assumed that the accelerometer is a low-pass system with an operating band limited by the cut-off frequency. Examples of the numerical values of the *UAE* for any time of accelerometer testing are presented in [17,18,19]. In addition, polynomial approximation was performed in [18] to determine the relationship between the error and the time of sensor testing. It was carried out using the Curve Fitting toolbox [22] built into MATLAB software. However, this type of approximation has a significant limitation due to the difficulties of determining the optimal order of the polynomial approximating the error [23,24]. Therefore, based on the Neural Network toolbox [25] built into MATLAB, the procedure for determining the optimal order of the polynomial approximating the error was presented in [26,27]. However, neither the structure nor the parameters of this polynomial were determined there. 

This paper proposes the use of the radial basis function neural network (*RBF-NN*) to determine the *RBFs* [28,29,30,31,32] for an assumed number of radial neurons. In this work, the *RBFs* were obtained using Python-related scientific packages that allow the easy and quick determination of the upper bound of absolute dynamic error. The error obtained based on the *RBF* is denoted below as the $UAE^{RBF}.$ The *RBFs* were determined on the basis of previously obtained values of the *UAE* for the assumed-in-advance ranges of variability of the accelerometer model parameters. Section 3 presents the mathematical models with associated descriptions of both the voltage-mode accelerometer and the model of the standard, which is the reference for determining the error. Based on these models, Section 4 presents a detailed description, along with the relevant mathematical relations of the algorithm used for determining the *UAE*. Then, Section 5 discusses the procedure used for determining the *RBF*.

The values of the accelerometer model parameters intended for substitution into the *RBF* can be assumed in advance or obtained as a result of the parametric identification of such a model. In order to precisely determine both the parameters of the model and the associated uncertainties, the identification procedure should be based on the measurement points of both frequency responses (amplitude and phase). For this purpose, it is convenient to use the weighted-least-squares (WLS) method discussed in detail in [33,34].

When the parameters and uncertainties are assumed-in-advance or obtained on the basis of the WLS method, it is not possible to apply the *RBF* directly because it is unclear for which values of the parameters from the ranges determined by the associated uncertainties the maximum error can be obtained. This maximum error is denoted below as the *UAE^RBF^*^(*max*)^. It is therefore necessary to use a parametric optimization method to determine this error. The solution of using the Monte Carlo (MC) method [34,35,36] based on a pseudorandom number generator with uniform distribution is discussed in detail in Section 6. It was convenient to employ here the Wichmann–Hill generator [37], which is recommended by the guide [34].

Section 7 presents the matrix containing the values of the *UAE*. These errors were determined for the case of changes of two parameters of a voltage-mode accelerometer for the assumed-in-advance ranges of these changes and quantization steps. Based on the matrix of errors, the optimal order, structure, and parameters values of the *RBF* were determined. The 5, 10, and 15 radial neurons were considered. Based on these functions, the error *UAE^RBF^* was determined for the selected values of the accelerometer parameters. Then, the values of uncertainties were assumed for the parameters and the *UAE^RBF^*^(*max*)^ values were calculated by employing the MC method.

The procedure for determining the *RBF* function as well as an application of the MC method to establish the error *UAE^RBF^* constitute the novelty of this paper.

## 2. General Guidelines for the Proposed Procedure

**Figure 1:**
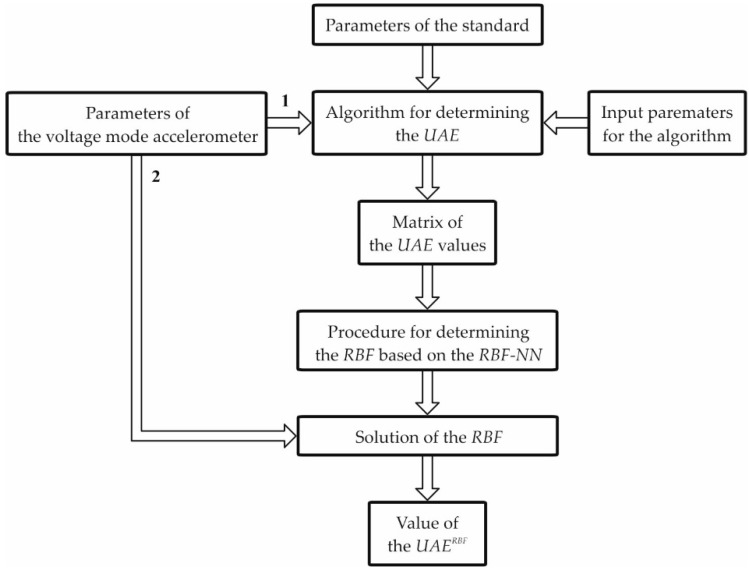
Block diagram of the procedure intended for determining the radial basis function (*RBF*) and the value of absolute dynamic error (*UAE^RBF^*).

Figure 1 shows the block diagram of the procedure intended for determining the *RBF* and then the value of *UAE^RBF^*.

The procedure above involves the algorithm for determining the *UAE*, which is presented in detail in Section 4. The input data for this algorithm are the parameters of the voltage-mode accelerometer and the standard, as well as the value of the signal limitation. The cut-off frequency of the standard was selected to be equal to the operational frequency bandwidth of the accelerometer. In the first step, denoted by 1 in Figure 1, the parameters of the voltage-mode accelerometer are generated with the assumed quantization steps and from the assumed ranges of their variability. The value of the *UAE* was determined for each set of a such parameters. In this way, the matrix of *UAE* values was obtained, which then serves as the input data for the procedure intended for determining the *RBF*. The procedure for determining the *RBF* based on the *RBF*-*NN* is discussed in detail in Section 5.

In the second step, denoted by 2, we can easily obtain the values of the error *UAE^RBF^* for the voltage-mode accelerometer by substitution of any set of parameters from the ranges assumed earlier. The *RBF*, therefore, allows the determination of the values of the *UAE^RBF^* without the need to use the procedures described in Section 4. If the values of parameters are known (without the associated uncertainties), then it is not necessary to carry out the parametric identification of the accelerometer model. Thus, the procedures described in Section 3 and Section 6 are also not necessary. However, it should always be kept in mind that the *RBF* is valid only for the ranges of variability of the accelerometer parameters for which it was determined earlier.

**Figure 2:**
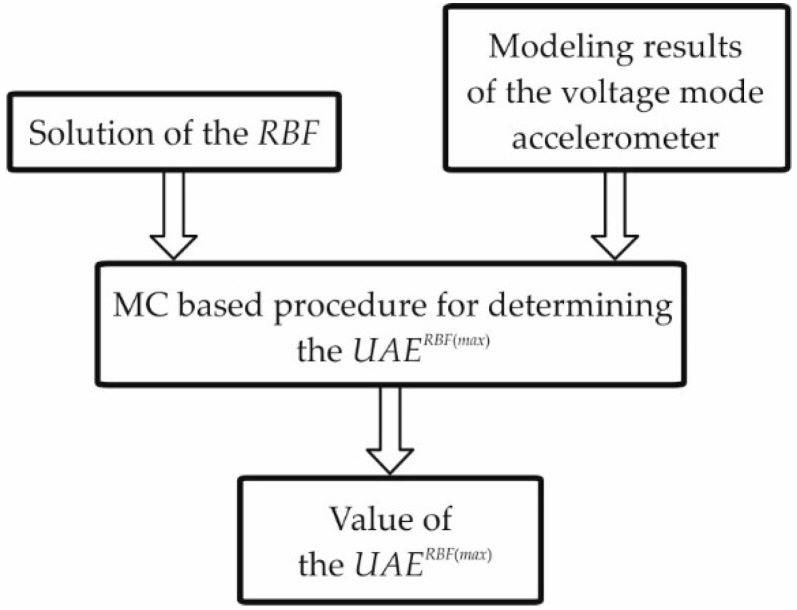
Block diagram of the procedure intended for determining the *UAE^RBF^*^(*max*)^.

For the assumed-in-advance ranges covering only the parameters obtained as a result of parametric identification (neglecting the uncertainties), the procedure shown in Figure 1 should be executed to determine the *RBF*. The block diagram of the procedure intended for determining the *UAE^RBF^*^(*max*)^ is shown in Figure 2.

The procedure above employs the previously determined *RBF*, as well as the parameters of the testing accelerometer and the associated uncertainties assumed-in-advance or obtained as a result of modeling carried out by the WLS method. This procedure is based on the MC method, which was employed to determine the values of accelerometer parameters from the ranges set by the associated uncertainties. As a result of implementation of this procedure, the *UAE^RBF^*^(*max*)^ was obtained. The MC-based procedure is presented in detail in Section 6. 

## 3. Mathematical Models of the Voltage-Mode Accelerometer and the Standard

The mathematical model of the voltage-mode accelerometer is most often represented by the transfer function:

(1) $$K_{V}\left(s\right)=\frac{-S_{V}\omega_{0}^{2}}{s^{2}+2\beta \omega_{0}s+\omega_{0}^{2}}$$

where

(2) $$\omega_{0}=2\pi f_{0}$$

while $S_{V},$ β, and $f_{0}$ are the voltage sensitivity $\left(V/{\mathrm{ms}}^{-2}\right),$ dimensionless damping ratio, and nondamped natural frequency (Hz), respectively.

The observer canonical form of the state-space representation associated with Equation (1) is

(3) $$K_{V}\left(s\right)=C_{V}{\left(sI_{1}-A_{V}\right)}^{-1}B_{V}$$

where I is the 2×2 dimensional identity matrix, while $A_{V},\;B_{V}$, and $C_{V}$ are

(4) $$A_{V}=\left[\begin{array}{cc} -2\beta \omega_{0} & 1\\ -\omega_{0}^{2} & 0 \end{array}\right],B_{V}=\left[\begin{array}{cc} 0 & -S_{V}\omega_{0}^{2} \end{array}\right],C_{V}=\left[\begin{array}{cc} 1 & 0 \end{array}\right].$$

Let the model of the standard with the cut-off frequency $f_{c}$ be given by the *K-*th order Butterworth filter:

(5) $$K_{s}\left(s\right)=\frac{n_{K}}{s^{K}+d_{1}\cdot s^{K-1}+d_{2}\cdot s^{K-2}+\ldots +d_{K-1}\cdot s+d_{K}}=\frac{S_{v}}{\prod_{k=1}^{K}\left[\frac{s}{2\pi f_{c}}-e^{\frac{j\left(2k+K-1\right)\pi }{2K}}\right]}.$$

The cut-off frequency $f_{c}$ of the standard is equal to the accelerometer operational frequency bandwidth.

The observer canonical form of the state-space representation related to the transfer Function (5) is

(6) $$K_{s}\left(s\right)=C_{s}{\left(sI_{2}\;-\;A_{s}\right)}^{-1}B_{s}$$

where

(7) $$A_{s}=\left[\begin{array}{cccccc} 0 & 1 & 0 & \ldots & 0 & 0\\ 0 & 0 & 1 & \ldots & 0 & 0\\ \vdots & \vdots & \vdots & \ddots & \vdots & \vdots \\ 0 & 0 & 0 & \ldots & 1 & 0\\ 0 & 0 & 0 & \ldots & 0 & 1\\ e & f & g & \ldots & h & i \end{array}\right],\;B_{s}={\left[\begin{array}{cccccc} 0 & 0 & \ldots & 0 & 0 & n_{K} \end{array}\right]}^{T},\;C_{s}={\left[\begin{array}{cccccc} 1 & 0 & \ldots & 0 & 0 & 0 \end{array}\right]}^{T}$$

and the variables in the last row of the matrix $A_{s}$ are $e=-d_{K}$, $f=-d_{K-1}$, $g=-d_{K-2}$, $h=-d_{2}$, and $i=-d_{1}.$ The matrix $I_{2}$ is the 6 ×6 dimensional matrix.

The difference between $K_{V}\left(s\right)$ and $K_{s}\left(s\right)$ is

(8) $$K\left(s\right)=K_{V}\left(s\right)-K_{s}\left(s\right)=C{\left(sI-A\right)}^{-1}B$$

where

(9) $$A=\left[\begin{array}{cc} A_{v} & 0\\ 0 & A_{s} \end{array}\right],B=\left[\begin{array}{c} B_{v}\\ B_{s} \end{array}\right],\;C=\left[\begin{array}{c} C_{v}\\ -C_{s} \end{array}\right],I=\left[\begin{array}{c} I_{1}\\ I_{2} \end{array}\right].$$

## 4. Algorithm for Determining the *UAE*

The upper bound of absolute dynamic error is determined by the following formula [12]:

(10) $$UAE=a\int_{0}^{T}\left|L^{-1}\left[K\left(s\right)\right]\right|dt=R_{L,L},\;L\epsilon \mathbb{N}$$

where a and T are the magnitude limitation of the input signal and the time of the accelerometer testing, respectively, while $L^{-1}$ denotes the inverse Laplace transformation.

The component $R_{L,L}$ in Equation (10) is the bottom-right element of the Romberg array and allows avoiding the numerical integration of the first component of UAE—Equation (10).

The Romberg array can be determined by

(11) $$R_{n,0}=a\left[R_{n,m-1}+\frac{1}{4^{m}-1}\left(R_{n,m-1}-R_{n-1,m-1}\right)\right],n,\;m=1,2,\ldots ,L$$

where

(12) $$R_{0,0}=0.5\cdot \left\{\left|L^{-1}{\left[K\left(s\right)\right]}_{t=0}\right|+\left|L^{-1}{\left[K\left(s\right)\right]}_{t=T}\right|\right\}$$

and

(13) $$R_{n,0}=0.5\cdot R_{n-1,0}+\frac{T}{2^{L}}\sum_{p=1}^{2^{L-1}}\left|k\left[2p-1\right]\frac{T}{2^{L}}\right|.$$

The value of L is determined by the stop condition for the Romberg method. 

The signal producing the error UAE is

(14) $$x_{A}\left(t\right)=a\cdot sgn\left[L^{-1}{\left[K\left(s\right)\right]}_{t=T-t}\right]$$

where sgn denotes the signum operation [12].

## 5. Procedure for Determining the *RBF* Based on the *RBF-NN*

The *RBF-NN* was proposed as a formal tool for mathematical modeling of error space [28,29,30,31,32]. The classical network architecture is applied where its structure consists of three layers: An input layer, a hidden layer with a nonlinear two-dimensional *RBF* activation function, and a linear output layer. This type of the network is characterized by the overall response function:

(15) $$RBF\left(x\right)\;=\;\overset{P}{\underset{p=1}{\sum }}a_{p}\cdot \varphi \left(||x-c_{p}||\right)$$

where P denotes the number of radial neurons, while $a_{p}$, $c_{p},$ and φ are the inner parameters and function, respectively.

The Gaussian kernel as the nonlinear *RBF* is presented as

(16) $$\varphi \left(||x-c_{p}||\right)=e^{\left[-\gamma ||x-c_{p}||{}^{2}\right]}$$

where γ and ${||\cdot ||}^{2}$ denote the inner parameter and squared Euclidian distance, respectively.

Given the assumptions, the *RBF* network can be treated as a universal approximator [28,29,30,31,32]. This means that such a system with a sufficient number of neurons is able to approximate any continuous function on a closed and bounded dataset with arbitrary precision. Here, the aim is to map the multivariate function of two arguments as follows:

(17) $$f:R^{2}\to R.$$

Hence, the network includes the input layer of size two and a single output. The hyperparameters $a_{p}$, $c_{p}$, and γ must be determined in a way that optimizes the match between φ and the given data. For model simplification, the parameter γ is fixed as the same for every Gaussian kernel function existing in the hidden layer.

The equations describing the approximator model can be presented in the form of a matrix notation as below:

(18) Gw=b

where the p×q dimensional matrix and the vectors denoted as G, w, and b respectively, have the following structure

(19) $$\left[\begin{array}{ccc} g_{11} & \ldots & g_{1P}\\ \vdots & \ddots & \vdots \\ g_{q1} & \ldots & g_{qP} \end{array}\right]\left[\begin{array}{c} w_{1}\\ \vdots \\ w_{p} \end{array}\right]=\left[\begin{array}{c} b_{1}\\ \vdots \\ b_{q} \end{array}\right]$$

where p and q denote the number of *RBF* neurons in the hidden layer and the number of input samples, respectively.

The elements of matrix G are the values of the *RBFs* evaluated at the points indicated by the input data according to the formula

(20) $$x_{j}:g_{ji}\;=\;\varphi \left(||x_{j}-c_{i}||\right)$$

where $x_{j}\in R^{2}$ are samples of the input data and $c_{i}\in R^{2}$ are centers of *RBF* for individual neurons. In turn, the vector b consists of the values of the original function known in the finite number of points, such that

(21) $$f\left(x_{i}\right)=b_{i}$$

and the values of the linear output weights are stored as the vector w.

Finally, the following training scheme was used to obtain the network hyperparameters relevant to the correct approximation:

1. The *RBF* centers were randomly sampled among the domain of the input dataset.
2. The value of parameter γ was selected from the set range with a given step.
3. For every value of parameter γ, the appropriate weights were calculated using a pseudoinverse solution.
  After the *RBF* centers $c_{i}$ are fixed, the weights that minimize the error at the output can be directly computed using a linear pseudoinverse method:
  (22) $$w=G^{+}b$$
  where $G^{+}$ denotes the Moore–Penrose pseudoinverse of the matrix G [38,39].
4. The determination coefficient $\left(R^{2}\right)$ and the mean squared error (MSE) were calculated.
5. Steps 2–4 were repeated for all indicated γ ranges to find the hyperparameters which optimize the value of the coefficient $R^{2}.$

## 6. MC-Based Procedure for Determining the *UAE^RBF^*^(*max*)^

Let the variables ${\tilde{S}}_{V},$ ${\tilde{f}}_{0}$, and $\tilde{\beta }$ denote the parameters of the mathematical model of the voltage-mode accelerometer assumed in advance or determined based on the WLS method [33,34], while the variables $u\left({\tilde{S}}_{V}\right),$ $u\left({\tilde{f}}_{0}\right)$, and $u\left(\tilde{\beta }\right)$ are the uncertainties associated with these parameters. Also, let

(23) $$\begin{array}{cc} {\tilde{S}}_{V}{}^{u}={\tilde{S}}_{V}+u\left({\tilde{S}}_{V}\right),\; & {\tilde{S}}_{V}{}^{l}={\tilde{S}}_{V}-u\left({\tilde{S}}_{V}\right)\\ {\tilde{f}}_{0}{}^{u}={\tilde{f}}_{0}+u\left({\tilde{f}}_{0}\right),\; & {\tilde{f}}_{0}{}^{l}={\tilde{f}}_{0}-u\left({\tilde{f}}_{0}\right)\\ {\tilde{\beta }}^{u}=\tilde{\beta }+u\left(\tilde{\beta }\right),\; & {\tilde{\beta }}^{l}=\tilde{\beta }-u\left(\tilde{\beta }\right) \end{array}$$

where (u) and (l) denote the upper and the lower ranges of the parameter changes by the values of associated uncertainties. 

If the RBF was determined on the basis of the accelerometer model parameters for the assumed-in-advance ranges $\langle S_{V}{}_{-},S_{V}{}_{+}\rangle ,$ $\langle f_{0}{}_{-},f_{0}{}_{+}\rangle ,$ and $\langle \beta_{-},\beta_{+}\rangle ,$ and if the below conditions

(24) $$\begin{array}{cc} {\tilde{S}}_{V}{}^{u}\in \langle S_{V}{}_{-},S_{V}{}_{+}\rangle , & {\tilde{S}}_{V}{}^{l}\in \langle S_{V}{}_{-},S_{V}{}_{+}\rangle \\ {\tilde{f}}_{0}{}^{u}\in \langle f_{0}{}_{-},f_{0}{}_{+}\rangle , & {\tilde{f}}_{0}{}^{l}\in \langle f_{0}{}_{-},f_{0}{}_{+}\rangle \\ {\tilde{\beta }}^{u}\in \langle \beta_{-},\beta_{+}\rangle , & {\tilde{\beta }}^{l}\in \langle \beta_{-},\beta_{+}\rangle \end{array}$$

are met, then it is possible to use the MC method to determine such values of the accelerometer model parameters ${\tilde{S}}_{V}{}^{max},$ ${\tilde{f}}_{0}{}^{max}$, and ${\tilde{\beta }}^{max}$ from the ranges $\langle {\tilde{S}}_{V}{}^{l},{\tilde{S}}_{V}{}^{u}\rangle ,$ $\langle {\tilde{f}}_{0}{}^{l},{\tilde{f}}_{0}{}^{u}\rangle$, and $\langle {\tilde{\beta }}^{l},{\tilde{\beta }}^{u}\rangle ,$ respectively, for which the value of *UAE^RBF^*^(*max*)^ is obtained. It is carried out on the basis of $RBF\left({\tilde{S}}_{V}{}^{max},{\tilde{f}}_{0}{}^{max},{\tilde{\beta }}^{max}\right)$.

**Figure 3:**
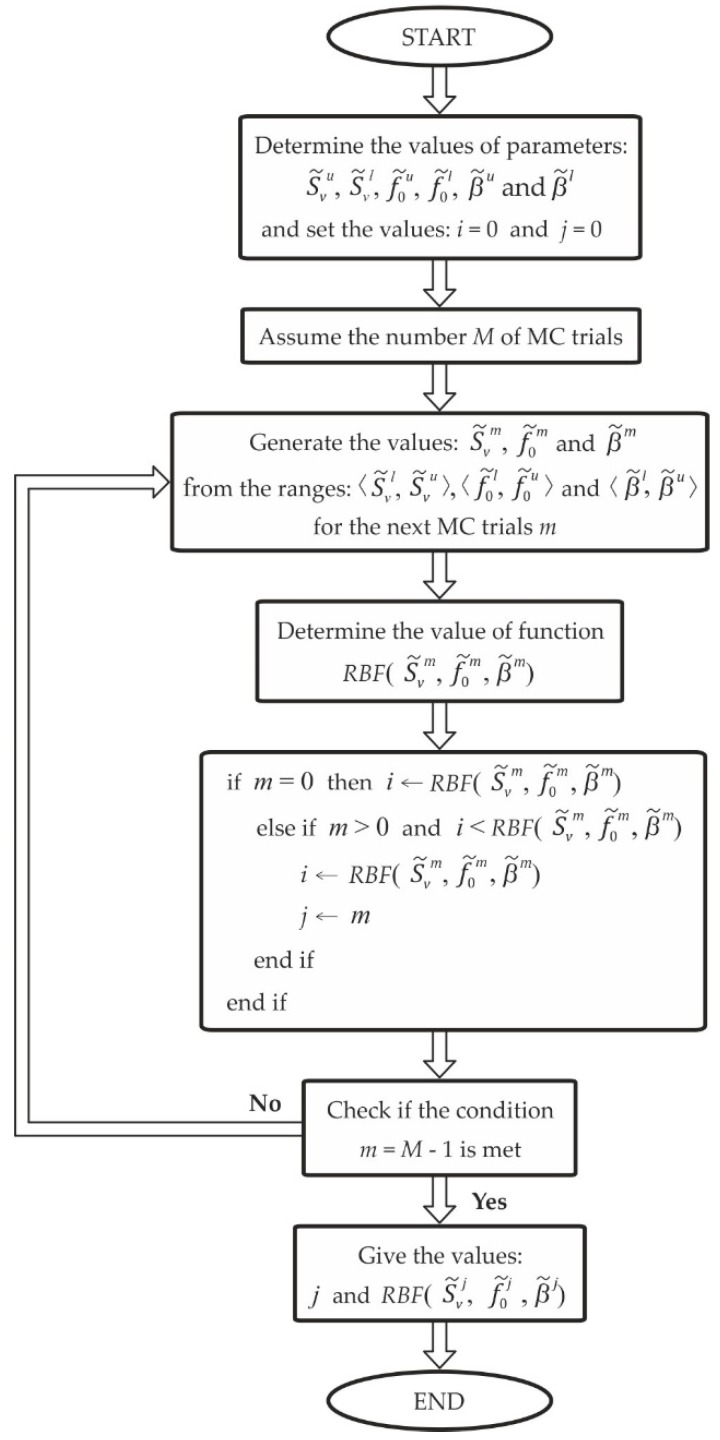
Block diagram of the Monte Carlo (MC)-based procedure for determining the *UAE^RBF^*^(*max*)^.

Figure 3 shows the block diagram of the MC-based procedure intended for determining the *UAE^RBF^*^(*max*)^. The lower number M of the MC trials is calculated based on the formula

(25) $$M>10^{4}/\left(1-p\right)$$

according to the guide [34], where p is the assumed coverage probability. The value of p is usually taken as equal to 0.95. During each MC trial (m=0,…M−1), the values of parameters ${\tilde{S}}_{V}{}^{m},$ ${\tilde{f}}_{0}{}^{m}$, and ${\tilde{\beta }}^{m}$ are generated from the ranges $\langle {\tilde{S}}_{V}{}^{l},{\tilde{S}}_{V}{}^{u}\rangle ,$ $\langle {\tilde{f}}_{0}{}^{l},{\tilde{f}}_{0}{}^{u}\rangle ,$ and $\langle {\tilde{\beta }}^{l},{\tilde{\beta }}^{u}\rangle ,$ respectively. Based on them, the following value of $RBF({\tilde{S}}_{V}{}^{m},$ ${\tilde{f}}_{0}{}^{m}$, ${\tilde{\beta }}^{m}$) is determined. The current maximum value of this function and the corresponding number m of trials are stored in memory (carried out by an assignment of these values to the variables i and j, respectively). For the trial equal to M−1, the value of $RBF({\tilde{S}}_{V}{}^{j},$ ${\tilde{f}}_{0}{}^{j}$, ${\tilde{\beta }}^{j}$) corresponding to the $UAE^{RBF\left(max\right)}$ is determined. The parameters ${\tilde{S}}_{V}{}^{j},$ ${\tilde{f}}_{0}{}^{j}$ and ${\tilde{\beta }}^{j}$ correspond to the parameters defined above by ${\tilde{S}}_{V}{}^{max},$ ${\tilde{f}}_{0}{}^{max}$ and ${\tilde{\beta }}^{max}.$

Uncertainties associated with the parameters ${\tilde{S}}_{V}{}^{j},$ ${\tilde{f}}_{0}{}^{j}$ and ${\tilde{\beta }}^{j}$ are determined based on the formula

(26) $$u\left(\delta \right)=\sqrt{\frac{1}{M-1}\overset{M-1}{\underset{m=0}{\sum }}{\left(\delta_{m}-\bar{\delta }\right)}^{2}}$$

where

(27) $$\bar{\delta }=\frac{1}{M}\overset{M-1}{\underset{m=0}{\sum }}\delta_{m}$$

and δ denotes the variable that should be substituted by this parameter of the accelerometer model for which the uncertainty is determined [11,34]. The uncertainty associated with the *UAE^RBF^*^(*max*)^ is determined in an analogous way (Figure 3).

## 7. Results and Verification

Table 1 includes the values of the UAE obtained based on the parameters from the ranges $S_{v}\in \langle 0.100,\;0.150\rangle$ and β∈〈0.0100, 0.0150〉, as well as for the constant value of parameter $f_{0}$ equal to 1 kHz.

The quantization steps for the parameters $S_{v}$ and β were equal to 0.002 and 0.0002, respectively. Taking into account the assumptions above, we have $S_{V}{}_{-}=0.100,$ $S_{V}{}_{+}=0.150,$ $\beta_{-}=0.0100$, and $\beta_{+}=0.0150$ according to Equation (38). The values of the *UAE* were obtained by utilizing the algorithm presented in Section 4 for the input parameters: $a=S_{v}$ and T=0.1 s. The 15th-order Butterworth filter with the cut-off frequency $f_{c}$ was determined by solving the equation describing the amplitude response obtained based on Equation (1).

On the basis of the values of the UAE tabulated in Table 1 and by applying the procedure presented in Section 5, the $RBF\left(S_{V},\beta \right)$ was determined. The cases of 5, 10, and 15 radial neurons based on Equations (28)–(30) were checked.

1. For five radial neurons:
  (28) $$\begin{array}{c} RBF_{5}\left(S_{V},\beta \right)=36131833920.92137e^{-0.020\left|{\left(0.0134-\beta \right)}^{2}+{\left(0.1400-S_{V}\right)}^{2}\right|}+\\ -11081182573.68980e^{-0.020\left|{\left(0.0114-\beta \right)}^{2}+{\left(0.1260-S_{V}\right)}^{2}\right|}47491590540.28882e^{-0.020\left|{\left(0.0146-\beta \right)}^{2}+{\left(0.1380-S_{V}\right)}^{2}\right|}+\\ -1065125193.52684e^{-0.020\left|{\left(0.0116-\beta \right)}^{2}+{\left(0.1100-S_{V}\right)}^{2}\right|}+23506064273.49600e^{-0.020\left|{\left(0.0148-\beta \right)}^{2}+{\left(0.1280-S_{V}\right)}^{2}\right|} \end{array}$$
2. For 10 radial neurons:
  (29) $$\begin{array}{c} RBF_{10}\left(S_{V},\beta \right)=-86057837.62482e^{-20\left|{\left(0.0124-\beta \right)}^{2}+{\left(0.1040-S_{V}\right)}^{2}\right|}+\\ -1137987870.37293e^{-20\left|{\left(0.0130-\beta \right)}^{2}+{\left(0.1220-S_{V}\right)}^{2}\right|}+3414860359.97698e^{-20\left|{\left(0.0128-\beta \right)}^{2}+{\left(0.1280-S_{V}\right)}^{2}\right|}+\\ -426811407.83125e^{-20\left|{\left(0.0142-\beta \right)}^{2}+{\left(0.1140-S_{V}\right)}^{2}\right|}-3260248505.26396e^{-20\left|{\left(0.0132-\beta \right)}^{2}+{\left(0.1280-S_{V}\right)}^{2}\right|}+\\ +172825594.36315e^{-20\left|{\left(0.0118-\beta \right)}^{2}+{\left(0.1420-S_{V}\right)}^{2}\right|}-1390178.49538e^{-20\left|{\left(0.0100-\beta \right)}^{2}+{\left(0.1060-S_{V}\right)}^{2}\right|}+\\ +415316898.45332e^{-20\left|{\left(0.0132-\beta \right)}^{2}+{\left(0.1100-S_{V}\right)}^{2}\right|}-243670439.6692e^{-20\left|{\left(0.0116-\beta \right)}^{2}+{\left(0.1400-S_{V}\right)}^{2}\right|}+\\ +1153163476.99674e^{-20\left|{\left(0.0138-\beta \right)}^{2}+{\left(0.1220-S_{V}\right)}^{2}\right|} \end{array}$$
3. For 15 radial neurons:
  (30) $$\begin{array}{c} RBF_{15}\left(S_{V},\beta \right)=50217932.38037e^{-50\left|{\left(0.0142-\beta \right)}^{2}+{\left(0.1360-S_{V}\right)}^{2}\right|}+\\ -338803095.53718e^{-50\left|{\left(0.0124-\beta \right)}^{2}+{\left(0.1160-S_{V}\right)}^{2}\right|}-99592530.74858e^{-50\left|{\left(0.0124-\beta \right)}^{2}+{\left(0.1040-S_{V}\right)}^{2}\right|}+\\ +1438055151.11707e^{-50\left|{\left(0.0126-\beta \right)}^{2}+{\left(0.1240-S_{V}\right)}^{2}\right|}-15000132.12083e^{-50\left|{\left(0.0100-\beta \right)}^{2}+{\left(0.1460-S_{V}\right)}^{2}\right|}+\\ -19539648.46192e^{-50\left|{\left(0.0148-\beta \right)}^{2}+{\left(0.1480-S_{V}\right)}^{2}\right|}+19065043.38108e^{-50\left|{\left(0.0138-\beta \right)}^{2}+{\left(0.1480-S_{V}\right)}^{2}\right|}+\\ +229678117.86818e^{-50\left|{\left(0.0110-\beta \right)}^{2}+{\left(0.1260-S_{V}\right)}^{2}\right|}+166603554.85264e^{-50\left|{\left(0.0132-\beta \right)}^{2}+{\left(0.1080-S_{V}\right)}^{2}\right|}+\\ -777217752.67323e^{-50\left|{\left(0.0124-\beta \right)}^{2}+{\left(0.1280-S_{V}\right)}^{2}\right|}-665634775.90997e^{-50\left|{\left(0.0120-\beta \right)}^{2}+{\left(0.1240-S_{V}\right)}^{2}\right|}+\\ -112930795.30634e^{-50\left|{\left(0.0150-\beta \right)}^{2}+{\left(0.1200-S_{V}\right)}^{2}\right|}+67735452.91154e^{-50\left|{\left(0.0110-\beta \right)}^{2}+{\left(0.1380-S_{V}\right)}^{2}\right|}+\\ +3949404.8155e^{-50\left|{\left(0.0102-\beta \right)}^{2}+{\left(0.1020-S_{V}\right)}^{2}\right|}+53414134.78681e^{-50\left|{\left(0.0110-\beta \right)}^{2}+{\left(0.1060-S_{V}\right)}^{2}\right|} \end{array}$$

Computational experiments were carried out for three given network structures with different hidden layer parameters and including sizes of 5, 10, and 15 neurons. Optimal hyperparameters and relevant criteria (statistical measures) regarding the quality of the model were calculated in each experiment. The obtained results are presented in Table 2, where Max error, *MSE*, *MAE*, *MedAE*, and *R*^2^ denote the maximum error, mean squared error, mean absolute error, median absolute error, and determination coefficient, respectively.

For the functions above, the coefficient $R^{2}$ was equal to 0.997300, 0.999970, and 0.999998, respectively. In turn, the values of *MSE* were: $1.27\times 10^{-4},$ $1.39\times 10^{-6}$, and $9.94\times 10^{-8},\;$respectively. For comparison, in the case of polynomial approximation presented in [18], the *MSE* was equal to 1.66 and 0.31, while in the case of using this approximation in [19], the fitting coefficient was equal to 0.797. Thus, it can easily be concluded that the fitting indexes obtained by the *RBF* are significantly better than those obtained with applying the polynomial approximation. In the case of 15 neurons, the values of *UAE^RBF^* obtained by substitution of the parameters $S_{V}$ and β from Table 1 into the $RBF_{15}\left(S_{V},\beta \right)$ were, in most cases, the same as the values of the *UAE* tabulated in this table.

**Figure 4:**
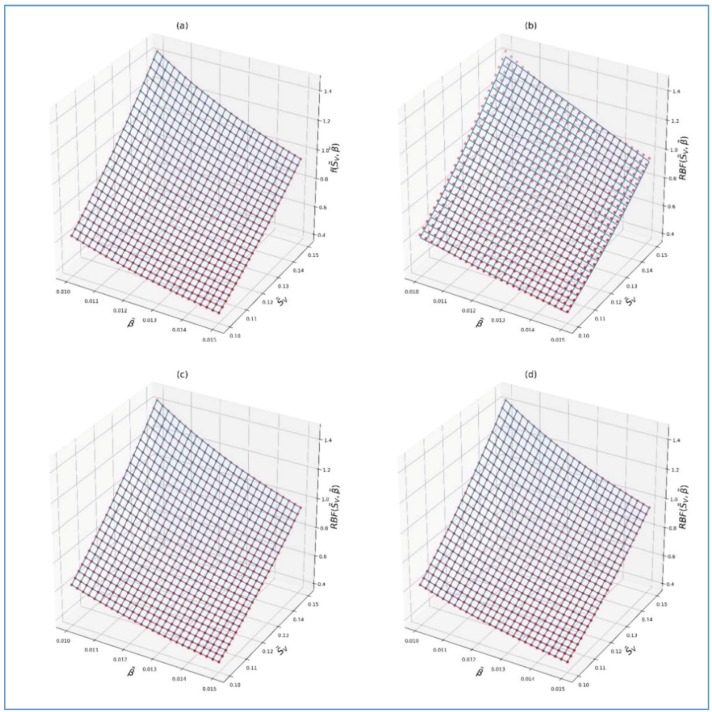
Visualization of the approximation surfaces obtained (blue wireframe), where original input dataset values are marked in red: (**a**) Original data, (**b**) surface mapped with 5 neurons, (**c**) surface mapped with 10 neurons, and (**d**) surface mapped with 15 neurons.

Figure 4a–d shows the values of the *UAE* tabulated in Table 1 and an approximation of the *UAE* using the *RBF* according to Equations (28)–(30).

The abovementioned approximation approach was implemented using Python 3.6 [40] and computed on a hardware configuration that included an Intel Core i5 M430, 2.27 GHz, 8 MB RAM and the operating system Windows 7 to obtain the experimental results. The following Python-related scientific packages were used in the implementation: NumPy, SciPy, iPython, Scikit-learn, Pandas, and Matplotlib for visualization purposes [41,42,43,44,45].

The values of *UAE^RBF^* determined based on $RBF_{15}\left(S_{V},\beta \right)$ for the values of parameters ${\tilde{S}}_{V}$ and $\tilde{\beta }$ selected from the ranges $\langle S_{V}{}_{-},S_{V}{}_{+}\rangle$ and $\langle \beta_{-},\beta_{+}\rangle$ are shown in Table 3.

Let us assume that the uncertainties associated with the parameters ${\tilde{S}}_{V}$ and $\tilde{\beta }$ included in Table 3 are: $u({\tilde{S}}_{V})=0.001$ and $u\left(\tilde{\beta }\right)=0.0001,$ respectively. In this case, according to Equation (23), we have ${\tilde{S}}_{V}{}^{u}=0.150,$ ${\tilde{S}}_{V}{}^{l}=0.100,$ ${\tilde{\beta }}^{u}=0.0150$, and ${\tilde{\beta }}^{l}=0.0100$. The conditions given by Equation (24) are therefore met. Hence, we can use the MC method based on the Wichmann–Hill pseudorandom number generator to determine the parameters ${\tilde{S}}_{V}{}^{max}$ and ${\tilde{\beta }}^{max}$, which produce the $UAE^{RBF\left(max\right)}.$ For p=0.95, the minimum number M of MC trials equal to $2\times 10^{5},$ obtained based on Equations (25), was applied for the calculations below.

The results of the MC simulation, tabulated in Table 4, are shown in the following order: *UAE^RBF^*^(*max*)^, ${\tilde{S}}_{V}{}^{max}\pm u({\tilde{S}}_{V}{}^{max}),$ ${\tilde{\beta }}^{max}\pm u({\tilde{\beta }}^{max})$, and the number m of the corresponding MC trial.

Based on the obtained results, it can be easily concluded that, in most cases, the values of parameters ${\tilde{S}}_{V}{}^{max}$ and ${\tilde{\beta }}^{max}$ were contained in the ranges $\langle S_{V}{}_{-},S_{V}{}_{+}\rangle$ and $\langle \beta_{-},\beta_{+}\rangle .$ It confirms the advisability of using the MC method to determine them. This is the only correct way to accurately determine the parameters of a voltage-mode accelerometer, which produce the $UAE^{RBF\left(max\right)}.$ When assuming the variability of all three parameters of the accelerometer, the functions of three variables are obtained, similar to those presented by Equations (28). When the values of parameters with associated uncertainties are obtained based on the WLS method, then the values of the parameters ${\tilde{S}}_{V}{}^{max},$ ${\tilde{f}}_{0}{}^{max}$, and ${\tilde{\beta }}^{max}$ and the value of *UAE^RBF^*^(*max*)^ are determined using the MC method in an analogous way to that above. 

## 8. Conclusions 

This paper presents the procedure for determining the *RBF* based on the numerical values of the *UAE* calculated for a voltage-mode accelerometer as an example. These *UAEs* were determined for the both the assumed-in-advance ranges of variability of the parameters of the accelerometer model and the quantization steps of these parameters. When the *RBF* is obtained in this way, we can easily and quickly calculate the *UAE* for any values of the parameters of an accelerometer model from the ranges above. The error obtained in this way is denoted by *UAE^RBF^*. The above facility results from the fact that it is not always necessary to use the algorithm dedicated to determinining the *UAE* and the related necessity of determining the cut-off frequency of the standard applied as a reference for calculating the error.

The paper also discussed in detail the use of the MC method to determine the *UAE* (such error is denoted by *UAE^RBF^*^(*max*)^) when considering the uncertainties associated with the parameters of an accelerometer model. To ensure the correct realization of the procedures for modeling a voltage-mode accelerometer by applying the parametric identification, such uncertainties should always be determined. The MC method is based on the previously determined *RBFs* for such ranges of change in the parameters of the accelerometer model, which contain all parameters for the accelerometer considered in a particular case. It is also important to underline that the parameters of such accelerometer, in the case of their decrease or increase by the values of the uncertainties associated with them, do not go beyond the lower and upper limits of the parameters for which the *RBF* was earlier determined.

The solutions presented in this paper regarding the determination of the *RBF* using an *RBF-NN* for an assumed number of radial neurons and the application of the MC method for determining the *UAE^RBF^*^(*max*)^ are the first solutions in the subject of measurement traceability. Based on the results obtained for the solutions above, in the case of 15 radial neurons, it can be seen that the statistical ratios regarding the uncertainty of approximation of the values of *UAE* using the *RBF* are much higher than those obtained using the polynomial approximation presented in [18] and [19]. The obtained values of these ratios also confirm that the number of neurons equal to 15 is optimal in terms of the uncertainty of an approximation of the *UAE* using the *RBF*.

The *RBFs* for the assumed range of variability of two accelerometer parameters were determined in this paper. This assumption was required to limit the number of calculation results intended for the presentation here. However, based on the displayed procedures, the *RBF* can be easily determined for the assumed ranges of variability of all three parameters of the accelerometer model.

Based on the obtained low uncertainty of the approximation for 15 neurons, it can be concluded that the *RBFs* determined for such a number of neurons can be successfully applied for the mutual comparison of the *UAE^RBF^*^(*max*)^ obtained for different types of accelerometers. It should be kept in mind, however, that the compared accelerometers should have the same frequency bandwidth of operation and that the parameters associated with their models must be within the ranges of parameter changes for which the *RBF* was determined.

## Figures and Tables

**Table 1:** Values of the *UAE*.

		β												
		**0.0100**	**0.0102**	**0.0104**	**0.0106**	**0.0108**	**0.0110**	**0.0112**	**0.0114**	**0.0116**	**0.0118**	**0.0120**	**0.0122**	**0.0124**
$S_{v}$	**0.100**	0.634	0.621	0.610	0.598	0.587	0.576	0.566	0.556	0.547	0.537	0.528	0.520	0.511
	**0.102**	0.660	0.647	0.634	0.622	0.611	0.600	0.589	0.579	0.569	0.559	0.550	0.541	0.532
	**0.104**	0.686	0.672	0.659	0.647	0.635	0.623	0.612	0.602	0.591	0.581	0.571	0.562	0.553
	**0.106**	0.712	0.698	0.685	0.672	0.659	0.648	0.636	0.625	0.614	0.604	0.594	0.584	0.575
	**0.108**	0.739	0.725	0.711	0.698	0.685	0.672	0.660	0.649	0.638	0.627	0.616	0.606	0.596
	**0.110**	0.767	0.752	0.738	0.724	0.710	0.697	0.685	0.673	0.661	0.650	0.639	0.629	0.619
	**0.112**	0.795	0.780	0.765	0.750	0.736	0.723	0.710	0.698	0.686	0.674	0.663	0.652	0.641
	**0.114**	0.824	0.808	0.792	0.777	0.763	0.749	0.736	0.723	0.710	0.698	0.687	0.675	0.664
	**0.116**	0.853	0.836	0.820	0.805	0.790	0.776	0.762	0.748	0.735	0.723	0.711	0.699	0.688
	**0.118**	0.883	0.865	0.849	0.833	0.817	0.803	0.788	0.774	0.761	0.748	0.736	0.724	0.712
	**0.120**	0.913	0.895	0.878	0.861	0.845	0.830	0.815	0.801	0.787	0.774	0.761	0.748	0.736
	**0.122**	0.943	0.925	0.907	0.890	0.874	0.858	0.843	0.828	0.813	0.800	0.786	0.773	0.761
	**0.124**	0.975	0.956	0.937	0.920	0.902	0.886	0.870	0.855	0.840	0.826	0.812	0.799	0.786
	**0.126**	1.006	0.987	0.968	0.950	0.932	0.915	0.899	0.883	0.868	0.853	0.839	0.825	0.812
	**0.128**	1.039	1.018	0.999	0.980	0.962	0.944	0.927	0.911	0.895	0.880	0.866	0.851	0.838
	**0.130**	1.071	1.050	1.030	1.011	0.992	0.974	0.957	0.940	0.924	0.908	0.893	0.878	0.864
	**0.132**	1.104	1.083	1.062	1.042	1.023	1.004	0.986	0.969	0.952	0.936	0.921	0.905	0.891
	**0.134**	1.138	1.116	1.095	1.074	1.054	1.035	1.016	0.999	0.981	0.965	0.949	0.933	0.918
	**0.136**	1.172	1.149	1.128	1.106	1.086	1.066	1.047	1.029	1.011	0.994	0.977	0.961	0.946
	**0.138**	1.207	1.183	1.161	1.139	1.118	1.098	1.078	1.059	1.041	1.023	1.006	0.990	0.974
	**0.140**	1.242	1.218	1.195	1.172	1.150	1.130	1.109	1.090	1.071	1.053	1.036	1.019	1.002
	**0.142**	1.278	1.253	1.229	1.206	1.183	1.162	1.141	1.121	1.102	1.083	1.065	1.048	1.031
	**0.144**	1.314	1.289	1.264	1.240	1.217	1.195	1.174	1.153	1.133	1.114	1.096	1.078	1.060
	**0.146**	1.351	1.325	1.300	1.275	1.251	1.229	1.207	1.185	1.165	1.145	1.126	1.108	1.090
	**0.148**	1.388	1.361	1.335	1.310	1.286	1.262	1.240	1.218	1.197	1.177	1.157	1.138	1.120
	**0.150**	1.426	1.398	1.372	1.346	1.321	1.297	1.274	1.251	1.230	1.209	1.189	1.169	1.150
		β												
		**0.0126**	**0.0128**	**0.0130**	**0.0132**	**0.0134**	**0.0136**	**0.0138**	**0.0140**	**0.0142**	**0.0144**	**0.0146**	**0.0148**	**0.0150**
$S_{v}$	**0.100**	0.503	0.495	0.488	0.480	0.473	0.466	0.459	0.453	0.447	0.440	0.434	0.428	0.423
	**0.102**	0.524	0.515	0.507	0.500	0.492	0.485	0.478	0.471	0.465	0.458	0.452	0.446	0.440
	**0.104**	0.544	0.536	0.528	0.520	0.512	0.504	0.497	0.490	0.483	0.476	0.470	0.463	0.457
	**0.106**	0.565	0.557	0.548	0.540	0.532	0.524	0.516	0.509	0.502	0.495	0.488	0.481	0.475
	**0.108**	0.587	0.578	0.569	0.560	0.552	0.544	0.536	0.528	0.521	0.514	0.507	0.500	0.493
	**0.110**	0.609	0.599	0.590	0.581	0.573	0.564	0.556	0.548	0.540	0.533	0.526	0.518	0.512
	**0.112**	0.631	0.621	0.612	0.603	0.594	0.585	0.576	0.568	0.560	0.552	0.545	0.537	0.530
	**0.114**	0.654	0.644	0.634	0.624	0.615	0.606	0.597	0.589	0.580	0.572	0.564	0.557	0.549
	**0.116**	0.677	0.667	0.656	0.646	0.637	0.627	0.618	0.609	0.601	0.593	0.584	0.577	0.569
	**0.118**	0.701	0.690	0.679	0.669	0.659	0.649	0.640	0.631	0.622	0.613	0.605	0.597	0.589
	**0.120**	0.725	0.713	0.702	0.692	0.681	0.671	0.662	0.652	0.643	0.634	0.625	0.617	0.609
	**0.122**	0.749	0.737	0.726	0.715	0.704	0.694	0.684	0.674	0.665	0.655	0.646	0.638	0.629
	**0.124**	0.774	0.762	0.750	0.739	0.728	0.717	0.706	0.696	0.687	0.677	0.668	0.659	0.650
	**0.126**	0.799	0.786	0.774	0.763	0.751	0.740	0.729	0.719	0.709	0.699	0.690	0.680	0.671
	**0.128**	0.824	0.812	0.799	0.787	0.775	0.764	0.753	0.742	0.732	0.722	0.712	0.702	0.693
	**0.130**	0.850	0.837	0.824	0.812	0.800	0.788	0.776	0.765	0.755	0.744	0.734	0.724	0.714
	**0.132**	0.877	0.863	0.850	0.837	0.824	0.812	0.801	0.789	0.778	0.767	0.757	0.747	0.737
	**0.134**	0.904	0.889	0.876	0.862	0.850	0.837	0.825	0.813	0.802	0.791	0.780	0.769	0.759
	**0.136**	0.931	0.916	0.902	0.888	0.875	0.862	0.850	0.838	0.826	0.815	0.803	0.793	0.782
	**0.138**	0.958	0.943	0.929	0.915	0.901	0.888	0.875	0.862	0.850	0.839	0.827	0.816	0.805
	**0.140**	0.986	0.971	0.956	0.941	0.927	0.914	0.901	0.888	0.875	0.863	0.851	0.840	0.829
	**0.142**	1.015	0.999	0.983	0.969	0.954	0.940	0.926	0.913	0.900	0.888	0.876	0.864	0.852
	**0.144**	1.043	1.027	1.011	0.996	0.981	0.967	0.953	0.939	0.926	0.913	0.901	0.888	0.877
	**0.146**	1.073	1.056	1.040	1.024	1.009	0.994	0.979	0.965	0.952	0.939	0.926	0.913	0.901
	**0.148**	1.102	1.085	1.068	1.052	1.036	1.021	1.006	0.992	0.978	0.965	0.951	0.939	0.926
	**0.150**	1.132	1.114	1.097	1.081	1.065	1.049	1.034	1.019	1.005	0.991	0.977	0.964	0.951

**Table 2:** Summary of model quality assessment for various hidden layer sizes.

Number of Neurons	Max Error (%)	*MSE*	*MAE*	*MedAE*	*R* ^2^
5	2.680	1.27 × 10^−4^	0.00940	0.00860	0.997300
10	0.310	1.39 × 10^−6^	0.00098	0.00093	0.999970
15	0.098	9.94 × 10^−8^	0.00024	0.00017	0.999998

**Table 3:** Values of the *UAE^RBF^* for the selected values of parameters ${\tilde{S}}_{V}$ and $\tilde{\beta }$.

		$\tilde{\beta }$			
		0.0101	0.0117	0.0133	0.0149
${\tilde{S}}_{V}$	**0.101**	0.641	0.552	0.487	0.434
	**0.117**	0.859	0.742	0.653	0.582
	**0.133**	1.11	0.959	0.843	0.753
	**0.149**	1.392	1.204	1.058	0.945

**Table 4:** Results of MC simulation.

		$\tilde{\beta }\pm u\left(\tilde{\beta }\right)$			
		0.01010±0.00001	0.01170±0.00001	0.01330±0.00001	0.01490±0.00001
${\tilde{S}}_{V}\pm u({\tilde{S}}_{V})$	0.1010±0.0001	0.643 $1011\cdot 10^{-4}\pm 3\cdot 10^{-9}$ $1009\cdot 10^{-5}\pm 4\cdot 10^{-11}$ 133,864	0.554 $1010\cdot 10^{-4}\pm 6\cdot 10^{-9}$ $1169\cdot 10^{-5}\pm 5\cdot 10^{-11}$ 95,693	0.488 $1011\cdot 10^{-4}\pm 7\cdot 10^{-9}$ $1329\cdot 10^{-5}\pm 8\cdot 10^{-11}$ 77,444	0.435 $1012\cdot 10^{-4}\pm 2\cdot 10^{-9}$ $1489\cdot 10^{-5}\pm 3\cdot 10^{-11}$ 47,515
	0.1170±0.0001	0.862 $1171\cdot 10^{-4}\pm 9\cdot 10^{-9}$ $1009\cdot 10^{-5}\pm 2\cdot 10^{-11}$ 17,202	0.744 $1171\cdot 10^{-4}\pm 7\cdot 10^{-9}$ $1169\cdot 10^{-5}\pm 3\cdot 10^{-11}$ 75,560	0.654 $1171\cdot 10^{-4}\pm 6\cdot 10^{-9}$ $1329\cdot 10^{-5}\pm 3\cdot 10^{-11}$ 23,843	0.584 $1171\cdot 10^{-4}\pm 3\cdot 10^{-9}$ $1489\cdot 10^{-5}\pm 3\cdot 10^{-11}$ 46,042
	0.1330±0.0001	1.110 $1131\cdot 10^{-4}\pm 2\cdot 10^{-9}$ $1009\cdot 10^{-5}\pm 5\cdot 10^{-11}$ 166,502	0.961 $1331\cdot 10^{-4}\pm 3\cdot 10^{-9}$ $1169\cdot 10^{-5}\pm 7\cdot 10^{-11}$ 194,645	0.845 $1331\cdot 10^{-4}\pm 1\cdot 10^{-9}$ $1329\cdot 10^{-5}\pm 4\cdot 10^{-11}$ 70,163	0.755 $1331\cdot 10^{-4}\pm 3\cdot 10^{-9}$ $1329\cdot 10^{-5}\pm 4\cdot 10^{-11}$ 175,809
	0.1490±0.0001	1.401 $1491\cdot 10^{-4}\pm 3\cdot 10^{-9}$ $1009\cdot 10^{-5}\pm 6\cdot 10^{-11}$ 181,360	1.210 $1491\cdot 10^{-4}\pm 2\cdot 10^{-9}$ $1169\cdot 10^{-5}\pm 5\cdot 10^{-11}$ 121,431	1.062 $1491\cdot 10^{-4}\pm 3\cdot 10^{-9}$ $1329\cdot 10^{-5}\pm 8\cdot 10^{-11}$ 76,186	0.947 $1491\cdot 10^{-4}\pm 4\cdot 10^{-9}$ $1489\cdot 10^{-5}\pm 2\cdot 10^{-11}$ 94,832
